# Bimetallic catalysis for C–C and C–X coupling reactions

**DOI:** 10.1039/c6sc05556g

**Published:** 2017-01-16

**Authors:** Dominic R. Pye, Neal P. Mankad

**Affiliations:** a Department of Chemistry , University of Illinois at Chicago , 845 W. Taylor St. , Chicago , IL 60607 , USA . Email: npm@uic.edu

## Abstract

Bimetallic catalysis represents an alternative paradigm for coupling chemistry that complements the more traditional single-site catalysis approach. In this perspective, recent advances in bimetallic systems for catalytic C–C and C–X coupling reactions are reviewed. Behavior which complements that of established single-site catalysts is highlighted. Two major reaction classes are covered. First, generation of catalytic amounts of organometallic species of *e.g.* Cu, Au, or Ni capable of transmetallation to a Pd co-catalyst (or other traditional cross-coupling catalyst) has allowed important new C–C coupling technologies to emerge. Second, catalytic transformations involving binuclear bond-breaking and/or bond-forming steps, in some cases involving metal–metal bonds, represent a frontier area for C–C and C–X coupling processes.

## Abstract 2

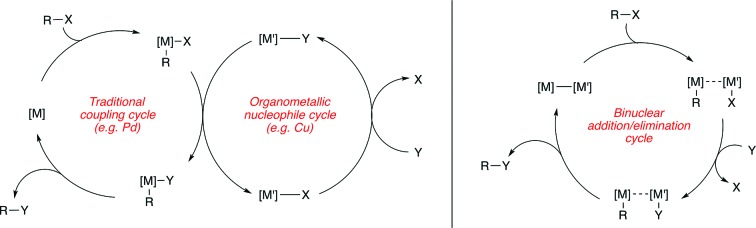
Bimetallic catalysis represents an alternative paradigm for coupling chemistry that complements the more traditional single-site catalysis approach.

## 1. Introduction

Coupling reactions that allow for catalytic C–C or C–X bond formation (X = *e.g.* B, N, O) have revolutionized synthetic chemistry by allowing complex organic structures to be created from simpler building blocks, even at late stages of multistep synthetic sequences.^1^–^5^ The dominant paradigm in coupling chemistry is to utilize single-site homogeneous catalysts and tailor reactivity and selectivity patterns using ligand design. For example, much of cross-coupling catalysis involves palladium–phosphine systems that operate by a canonical oxidative addition/reductive elimination cycling mechanism. Decades have been spent designing elaborate phosphine ligands to provide reactivity suitable for modern applications.^6^–^9^ Exquisite levels of catalytic activity, regioselectivity, and/or stereoselectivity ultimately have been achieved using this paradigm.

Pursuing alternative catalytic paradigms that go beyond this single-site approach has the potential to uncover complementary reactivity and selectivity regimes.^10^ In addition, in some cases catalytic reactivity can become accessible with inexpensive and earth-abundant metals not typically utilized extensively in cross-coupling catalysis. This perspective highlights one such alternative approach: the use of bimetallic catalysis for C–C and C–X bond forming reactions.^11^ The focus of the perspective is on recent developments in bimetallic catalysis as applied to catalytic C–C and C–X bond formation in molecular organic systems. All of the included examples involve catalytic amounts of two d-block metals cooperating during catalysis. The following topics are excluded from this perspective: reactions involving d-block metals cooperating with main-group elements (*e.g.* Na, Al, B, P) during catalysis;^12^,^13^ cases with one of the two metals not participating directly in bond-breaking/forming events (*e.g.* photoredox catalysis,^14^ Wacker oxidation^15^); cases with one of the two metals present in stoichiometric quantities; tandem, cascade, or domino reactions^16^ that do not involve direct communication between codependent catalytic metals; and cluster systems where mechanistic understanding is limited.^17^ Also excluded from the perspective are catalytic polymerization reactions,^18^–^20^ reductions of unsaturated organics (*e.g.* hydrogenation, hydrosilylation),^21^–^25^ and transformations of small-molecule inorganics (*e.g.* H_2_, H_2_O, CO, CO_2_, N_2_, O_2_, *etc.*),^26^–^30^ all of which have had recent advances in their own right using bimetallic approaches.

**Scheme 1:**
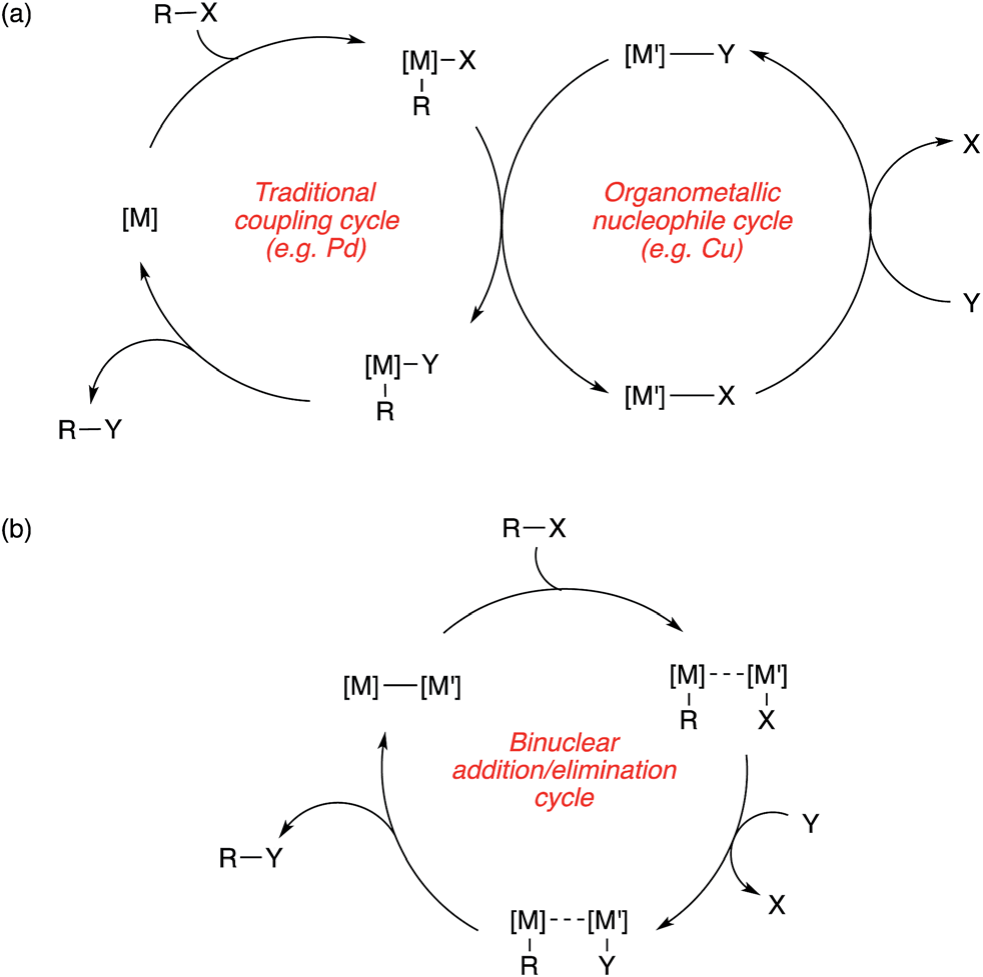
Representative mechanistic schemes for bimetallic catalysis (a) without and (b) with binuclear bond breaking and forming events.

The contribution of this perspective is timely, as bimetallic catalysis for C–C and C–X coupling is a burgeoning area that stands to make important contributions to the synthetic toolkit. In this perspective, these contributions are categorized into two broad subdivisions, with representative mechanistic schemes shown in Scheme 1. First are bimetallic systems involving catalytic generation of an organometallic nucleophile that undergoes transmetallation with a “traditional” coupling catalyst that operates using its canonical single-site mechanism (Scheme 1a). Here, no binuclear steps are utilized for breaking the bonds of the coupling partners or forming the bonds of the products.^31^ Second are bimetallic systems that involve binuclear bond activation and/or bond elimination events (Scheme 1b). Here, metal–metal bonds often (though not always) play key roles during catalysis.^32^

## 2. Bimetallic catalysis with mononuclear bond breaking & forming mechanisms

### 2.1 Organocopper nucleophiles

**Scheme 2:**
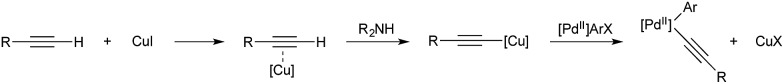
Cu/Pd cooperation in Sonogashira coupling.

It was demonstrated in 1966 by Owsley and co-workers that copper-acetylides react in a stoichiometric fashion with aryl halides.^33^ The harsh reaction conditions (reflux in pyridine) lead to the formation of side products, such as aryl halide reduction and acetylene dimerization. Monometallic, palladium-catalysed coupling of aryl halides and terminal alkynes was reported by both Cassar^34^ and Heck^35^ in 1975; however both catalyst systems still required elevated temperature for high conversion. In 1975 Sonogashira and co-workers reported that the addition of copper salts greatly accelerated the reaction, leading to room temperature reactivity, greater functional group tolerance, and a generally more useful procedure.^36^ It is now accepted that the role of copper in these reactions is to react with the alkyne in the presence of a base to give a copper acetylide.^37^ The acetylide moiety then undergoes transmetallation from copper to palladium to give the key arylpalladium(ii) acetylide (Scheme 2), which subsequently releases the desired product by reductive elimination. The key step, transmetallation of an organic fragment to palladium, has proven the inspiration for all the copper–palladium bimetallic-catalysis described in this section.

#### 2.1.1 Borylcupration and hydrocupration of unsaturated C–C bonds

Traditional cross-coupling requires the pre-synthesis of organometallic or organo-main group nucleophiles for use. Organometallic nucleophiles are often unstable, and require synthesis immediately prior to use. Organo-main group nucleophiles are usually bench stable, but also require synthesis and purification prior to use. In addition to these drawbacks, stoichiometric metal or main group byproducts are unavoidable in such couplings.

**Scheme 3:**
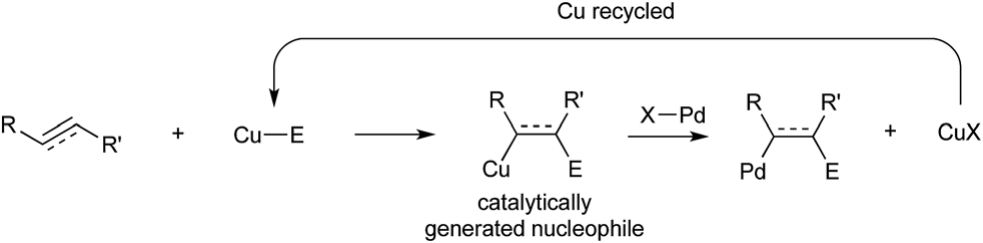
General scheme for organocopper nucleophiles generated by element-cupration.

An alternative mechanistic paradigm is the catalytic, *in situ* formation of organometallic species from catalytic amounts of a precatalyst and an organic pro-nucleophile. This avoids the pre-formation and purification of the nucleophilic component, and the organic pro-nucleophiles are generally more stable and readily available than organometallic or main group nucleophiles. One example of this approach is the insertion of unsaturated organic compounds into copper-element bonds (Scheme 3). The product of this insertion is a reactive organo-copper nucleophile ready for further reaction. A further advantage in catalytic formation of the nucleophile is that low concentrations of reactive species leads to fewer undesired side reactions. The functionalisation of an unsaturated bond also leads to further complexity build up in a single step.

**Scheme 4:**
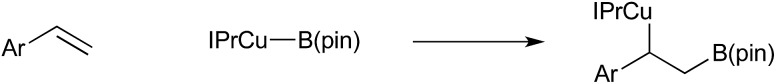
Borylcupration of styrenes demonstrated by Sadighi.

The insertion of alkenes in to copper–boron bonds has been implicated in many copper-catalysed C–B bond forming processes, such as copper-catalysed hydroboration of alkenes. In 2006 Sadighi proved this unambiguously by the isolation of β-boryl alkyl copper species from the reaction of IPrCu(Bpin) with styrenes (Scheme 4).^38^

**Scheme 5:**
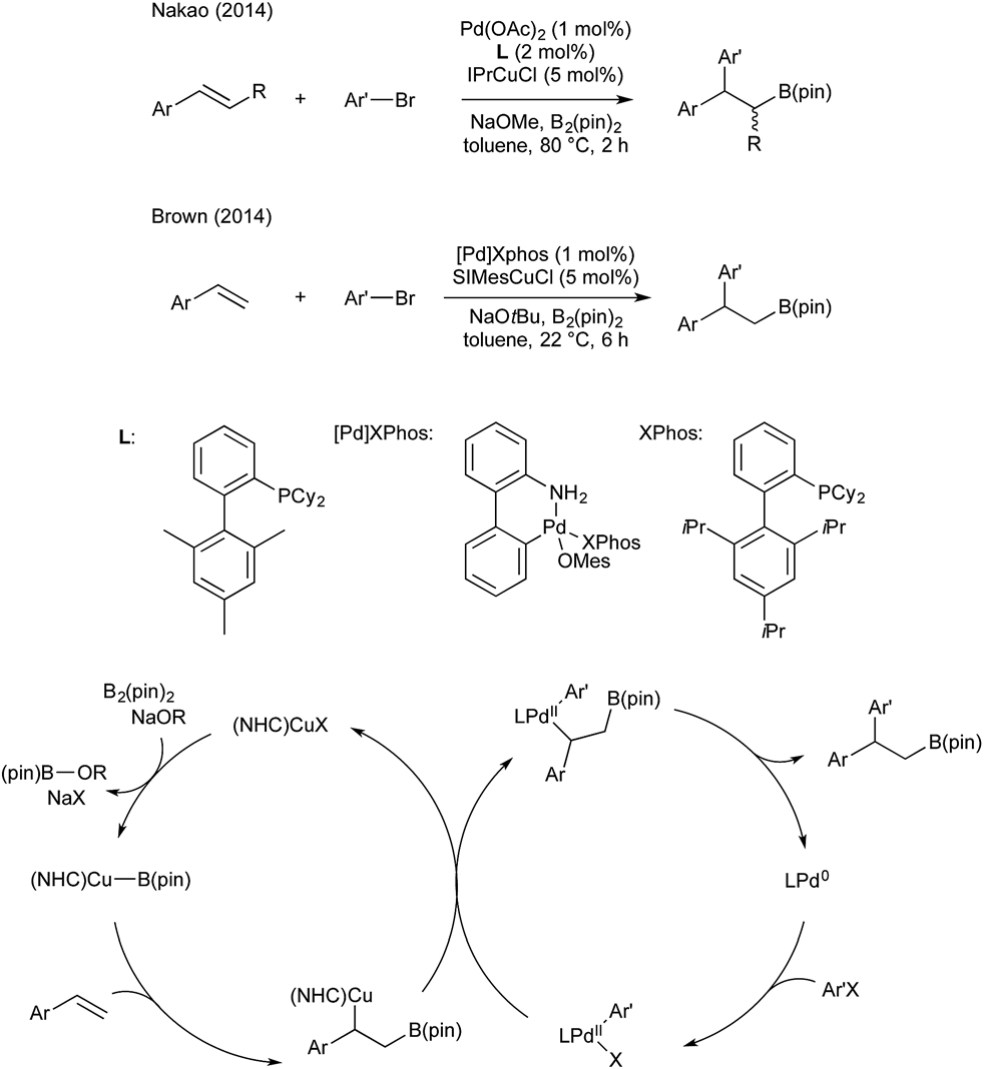
Cu/Pd catalysis for arylboration developed by the groups of Semba/Nakao and Brown.

In 2014 Semba/Nakao^39^ and Brown^40^ independently showed that this intermediate could be used as a transmetallating reagent from copper to an arylpalladium(ii), which reductively eliminates to form an carboborylated product and Pd(0). The copper boryl species is regenerated through alkoxide assisted transmetallation with B_2_(pin)_2_, and palladium(0) undergoes oxidative addition with an aryl bromide to complete two synergistic catalytic cycles. Both groups reported (NHC)copper(i) and Pd(ii)/dicyclohexylbiarylphosphine precatalyst mixtures (Scheme 5).

**Scheme 6:**
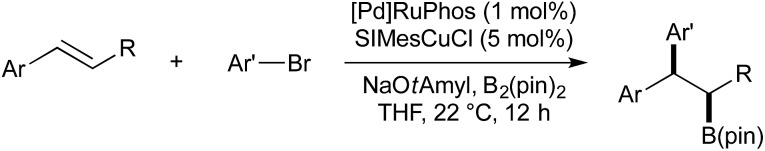
Diastereoselective carboboration developed by Brown.

Brown and co-workers also showed that when cyclic styrene derivatives (such as 1,2-dihydronapthalene) were employed as substrates, in most cases the reaction gave the *trans* diastereomer with high selectivity. As the addition of (SIMes)CuB(pin) across the alkene is likely to proceed in a *syn*-fashion, it was stated that the transmetallation Cu–Pd must proceed with inversion of stereochemistry to give the *trans* isomer after stereoretentive reductive elimination. Under the described conditions, acyclic, 1,2-disubstituted styrenes gave low diastereoselectivity. Brown later reported modified conditions under which both the *syn*- and *anti*-carboboration diastereomers of acyclic disubstituted styrenes could be obtained, selectivity being determined by a change in solvent and ligand (Scheme 6).^41^ It was found that the use of THF and RuPhos would selectively provide the *syn*-product, presumably through a stereoretentive transmetallation of the putative *syn*-Cu-alkyl intermediate, followed by reductive elimination. Diastereoselectivity was found to be reversed if the solvent was changed to toluene, and the Pd ligand to triisobutylphosphine. It should be noted that both the change of solvent and ligand were required; both Pd-RuPhos in toluene and Pd-PiBu_3_ in THF gave low diastereoselectivity.

**Scheme 7:**
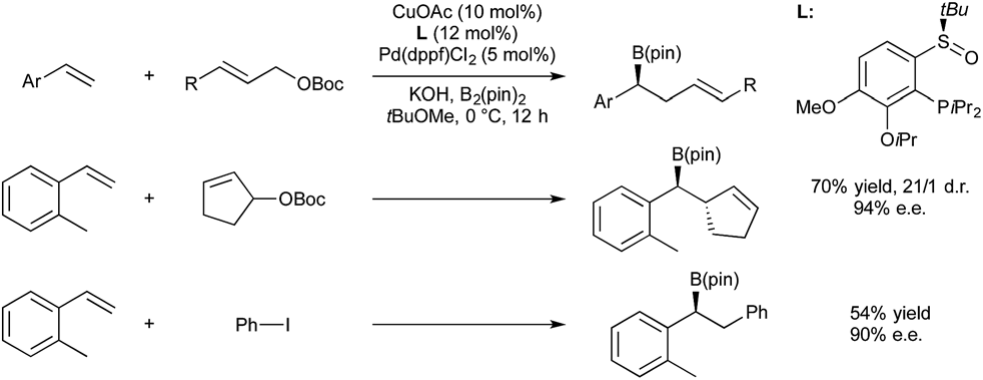
Enantioselective carboboration developed by Liao.

Liao and co-workers reported an enantioselective variant of the Cu/Pd carboboration reaction in 2015 (Scheme 7).^42^ Here they use copper(i) acetate with a chiral sulfoxide–phosphine ligand to achieve enantioselective borylcupration of a styrene, followed by transmetallation to a palladium-allyl species generated from the oxidative addition of allyl-*tert*-butylcarbonates to Pd(0). Products were generated with ee > 90%. When racemic, cyclic allylic carbonates were used as electrophiles, diastereomeric control of the two contiguous chiral centres was achieved. It was also demonstrated that iodobenzene could be used in place of the allylic carbonate to give an enantioselective version of the Nakao–Brown carboboration, albeit in lower yield.

**Scheme 8:**
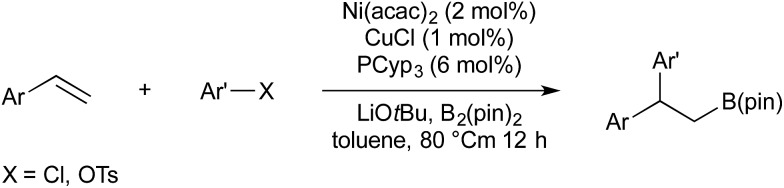
Cu/Ni catalysis developed by Semba/Nakao.

In 2016 Semba/Nakao reported a procedure for the carboboration of alkenes, this time using nickel rather than palladium to activate the aryl electrophile.^43^ As a first-row transition metal, nickel is preferable to palladium in terms of cost and earth abundance. It was also found that the Nakao–Brown conditions were not suitable for the use of aryl chlorides, or phenol derived electrophiles (other than triflates). Semba/Nakao demonstrated that styrenes could be converted to their carboborylated product using a precatalyst system of CuCl, Ni(acac)_2_, and tricyclopentylphosphine, whilst using aryl chlorides or tosylates as the electrophilic carbon source (Scheme 8). Drawbacks, in comparison with the copper/palladium-catalysed systems are the requirement for higher temperatures (80 °C *vs.* room temperature) and the poor reactivity of β-substituted styrenes (30–40% yield).

**Scheme 9:**
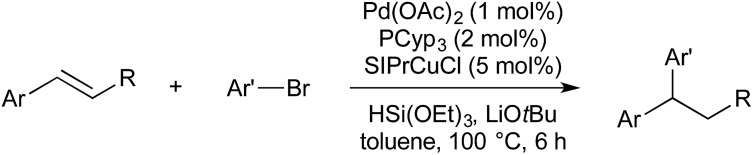
Hydroarylation catalysis developed by Semba/Nakao.

In 2016 Semba/Nakao reported a system wherein borylcupration could be replaced with hydrocupration, thereby furnishing 1,1-diarylalkanes (Scheme 9).^44^ (NHC)CuH is generated *in situ* by the reaction of (NHC)CuOtBu with HSi(OEt)_3_. The use of a deuterium labelled silane gave confirmation of *syn*-addition across the double bond, and subsequent stereoinversion upon transmetallation. Theoretical calculations suggest an S_E_2(back) mechanism, which is consistent with stereoinversion.

**Scheme 10:**
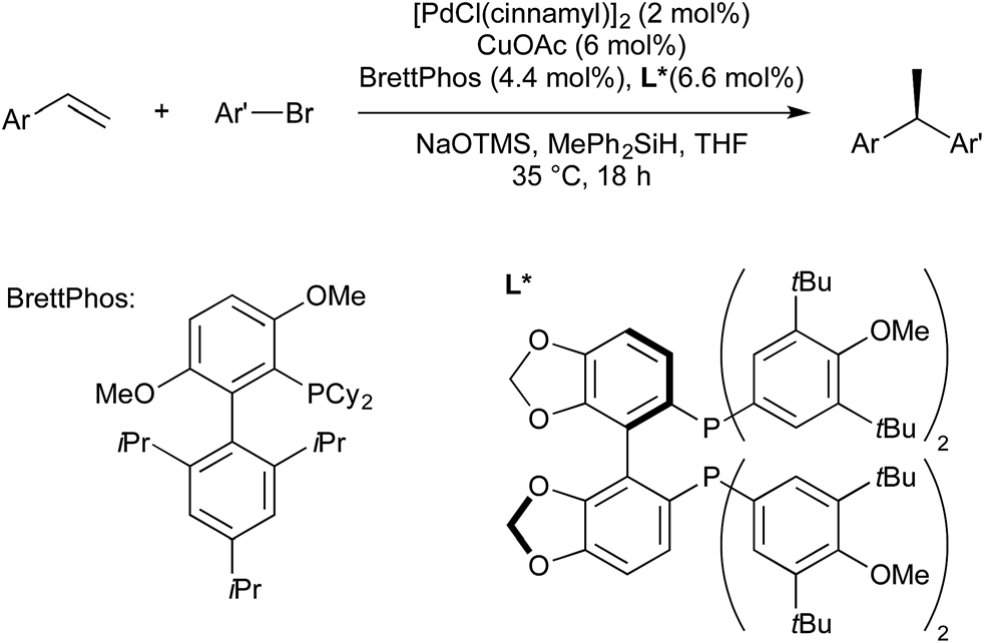
Enantioselective hydroarylation developed by Buchwald.

Later that year Buchwald used a chiral bisphosphine Cu(i) precatalyst to give an enantioselective Cu/Pd hydroarylation of styrenes (Scheme 10).^45^ The enantiodetermining step is the addition of a chiral copper hydride across the double bond.

**Scheme 11:**
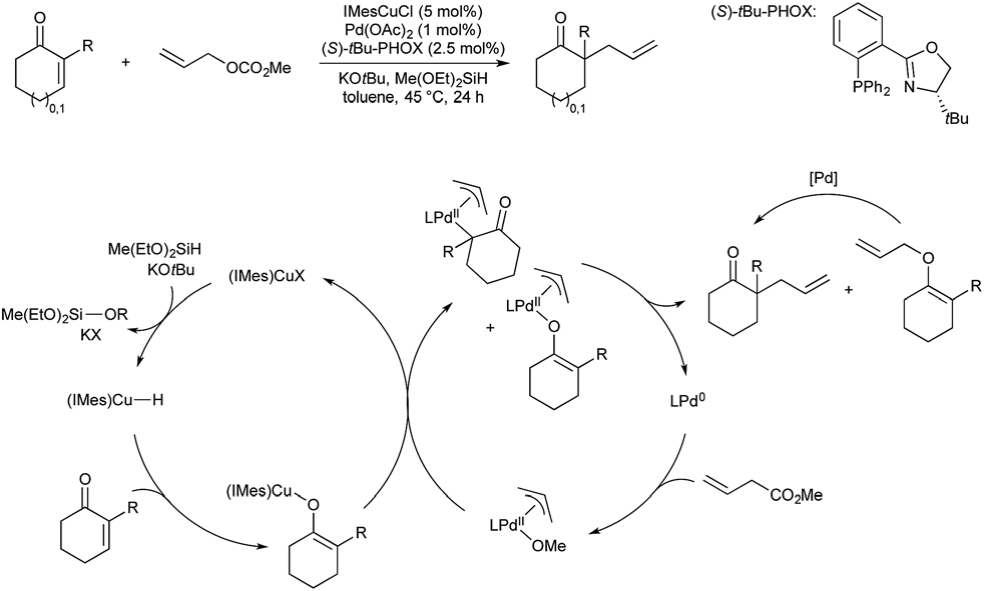
Reductive allylation of α,β-unsaturated ketones developed by Riant.

Riant and co-workers have described an asymmetric reduction/allylation of α,β-unsaturated ketones using a copper–palladium bimetallic catalyst system, a silane, and allyl carbonates.^46^ The products of these reactions are highly valuable, chiral all-carbon quaternary centres. In this instance the enantioselective step is governed by the palladium catalyst, bearing a chiral PHOX ligand, as opposed to a chiral copper hydride *vide supra*. Copper-catalysed 1,4-reduction of the unsaturated ketone gives a copper-enolate, which will then transmetallate to palladium. Mechanistic studies suggest that this transmetallation can give both the *C*- and *O*-bound Pd-enolate, which, after reductive elimination, release both the desired product and an allyl-enol ether. The allyl-enol ether converts to the desired product under the reaction conditions *via* a palladium-catalysed Cope rearrangement (Scheme 11).

**Scheme 12:**
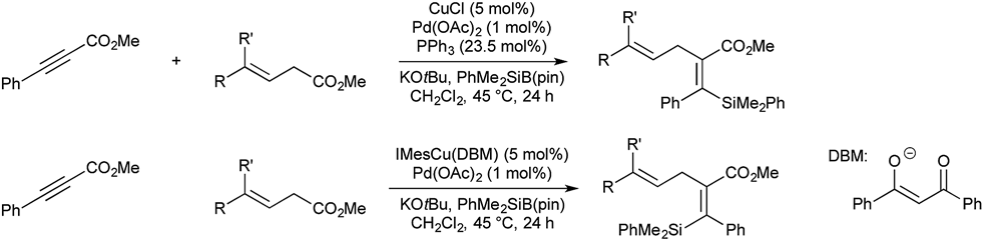
Silylation catalysis developed by Riant.

The reaction of copper(i) complexes with silyl boranes is known to give copper-silyl species, which Riant and co-workers showed will add across electron deficient alkynes to catalytically generate a vinyl-copper species to be used in palladium-catalysed cross-coupling (Scheme 12).^47^ In this instance allylic carbonates were used as the electrophiles. The authors found that the regioselectivity of the double bond could be controlled, switching between the *Z*- and *E*-isomers by omitting triphenylphosphine from the reaction conditions, and switching from simple copper chloride to NHC ligated IMesCu(DBM) (DBM = dibenzoylmethanate).

The effect of triphenyl phosphine on the regioselectivity is ascribed to the relative reactivity of the palladium(ii) allyl present, and the steric hindrance of the initial *syn*-silylcupration product compared to its tautomeric allenolate. In the absence of phosphine, palladium dimer [Pd(allyl)Cl]_2_ forms. Transmetallation from copper gives an allyl-, vinyl-palladium intermediate from which C–C bond formation occurs *via* an inner-sphere mechanism. In the presence of excess phosphine, cationic [(Ph_3_P)_*n*_Pd(allyl)]^+^ forms, which does not transmetallate with copper. In this instance the less sterically hindered allenolate reacts with the Pd-allyl complex *via* an outer-sphere mechanism, giving the products of formal anti-addition.

#### 2.1.2 C–C cross-coupling

**Scheme 13:**
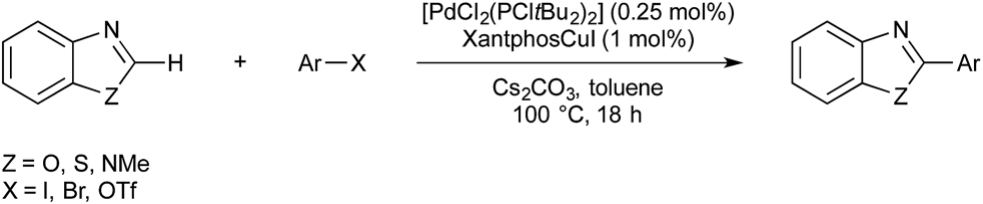
Cu/Pd cross-coupling developed by Faul.

Faul and co-workers demonstrated a C–H activation/biaryl coupling, catalysed by copper and palladium (Scheme 13).^48^ In this instance an organocopper intermediate is formed by the C–H activation of pharmaceutically privileged benzo-thiozoles, -oxazoles and -imidazoles. This then acts as a nucleophilic coupling partner for palladium-catalysed cross-coupling with an aryl halide. A related example was reported by Cazin.^49^

The use of tri(organo)silanol nucleophiles in C–C cross-coupling (Hiyama–Denmark coupling) is an attractive alternative to Sn, Mg, Zn, Al, and B based nucleophiles due to the bench stability and low toxicity of silicon compounds. Whilst monometallic systems have been shown to successfully couple C(sp^2^)–silicon nucleophiles with aryl halides and sulfonates, no general procedure has been developed for the coupling of alkyl-silicon nucleophiles with aryl sulfonates.

**Fig. 1:**
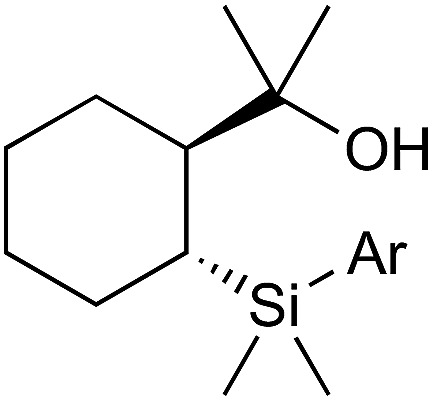
Alkylsilane reagent developed by Nakao.

The Nakao group have developed aryl[2-(2-hydroxyprop-2-yl)cyclohexyl]dimethylsilanes (Fig. 1) for nickel-catalysed biaryl coupling with aryl sulfonates.^50^ This reaction, however, suffers from poor tolerance of sensitive functional groups and sterically demanding substrates.

**Scheme 14:**
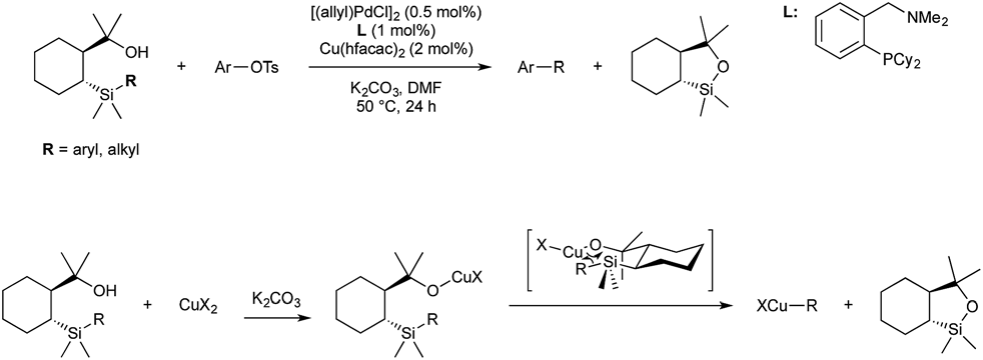
Cu/Pd variant of Hiyama–Denmark coupling developed by Nakao.

In 2016 Nakao published a revised procedure consisting of a bimetallic, Cu/Pd catalyst system which displays much greater functional and steric compatibility, as well as being compatible with alkyl[2-(2-hydroxyprop-2-yl)cyclohexyl]dimethylsilanes (Scheme 14).^51^

In this reaction the difunctional silyl-alcohol reagent initially coordinates to copper through deprotonation/salt metathesis of the alcohol, followed by intramolecular [2 + 2] σ-bond metathesis to give an organocopper intermediate (Scheme 14). This transmetallates to arylpalladium(ii) (from Pd(0) and aryl tosylate). Reductive elimination furnishes the product and closes the dual catalytic cycle.

#### 2.1.3 Modular imine synthesis

**Scheme 15:**
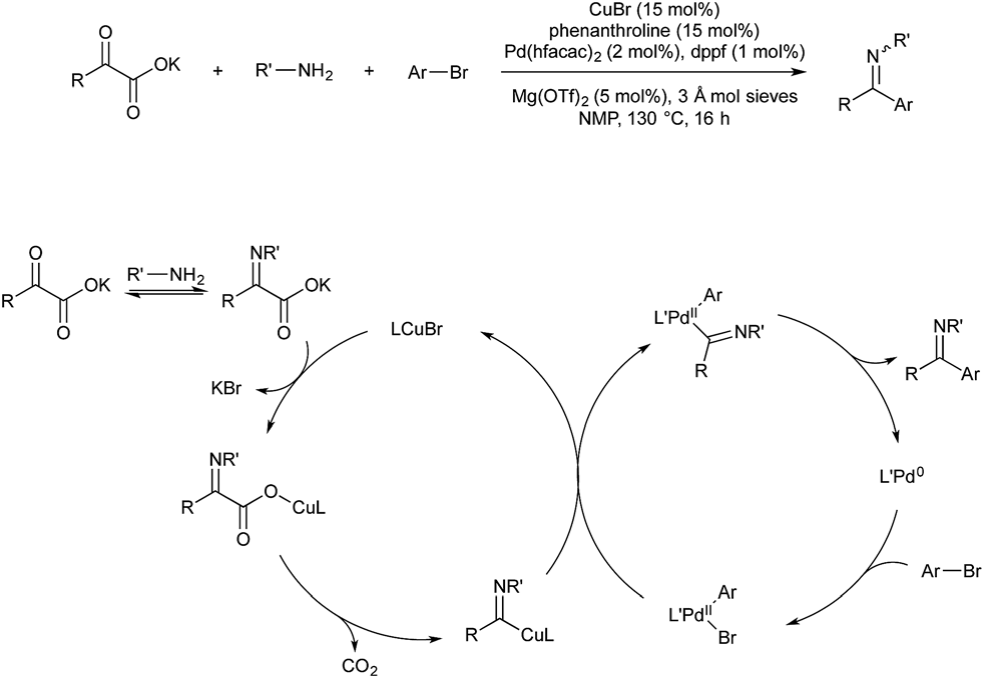
Cu/Pd-catalyzed imine synthesis.

Imines are highly desirable synthetic intermediates due to the high number of functional group transformations and C–C bond forming reactions that they can undergo. Traditional methods for their synthesis, such as Friedel–Crafts or organometallic additions to nitriles require harsh conditions and display poor functional group compatibility. Condensation of ketones with amines, whilst proceeding under mild conditions, does not allow for one pot, fully modular synthesis, as both R_1_ and R_2_ must be already defined in the ketone starting material. Goossen and co-workers have described a modular, three-component coupling of amines, α-ketocarboxylates and aryl halides catalysed by copper and palladium.^52^,^53^ *In situ* formation of an α-iminocarboxylate by amine-carbonyl condensation is followed by salt metathesis with copper-bromide. Decarboxylation provides an iminoacyl-copper species which transmetallates to Pd(ii) aryl. Reductive elimination provides the desired imine and renders the cycle catalytic (Scheme 15).

### 2.2 Organogold nucleophiles

**Scheme 16:**
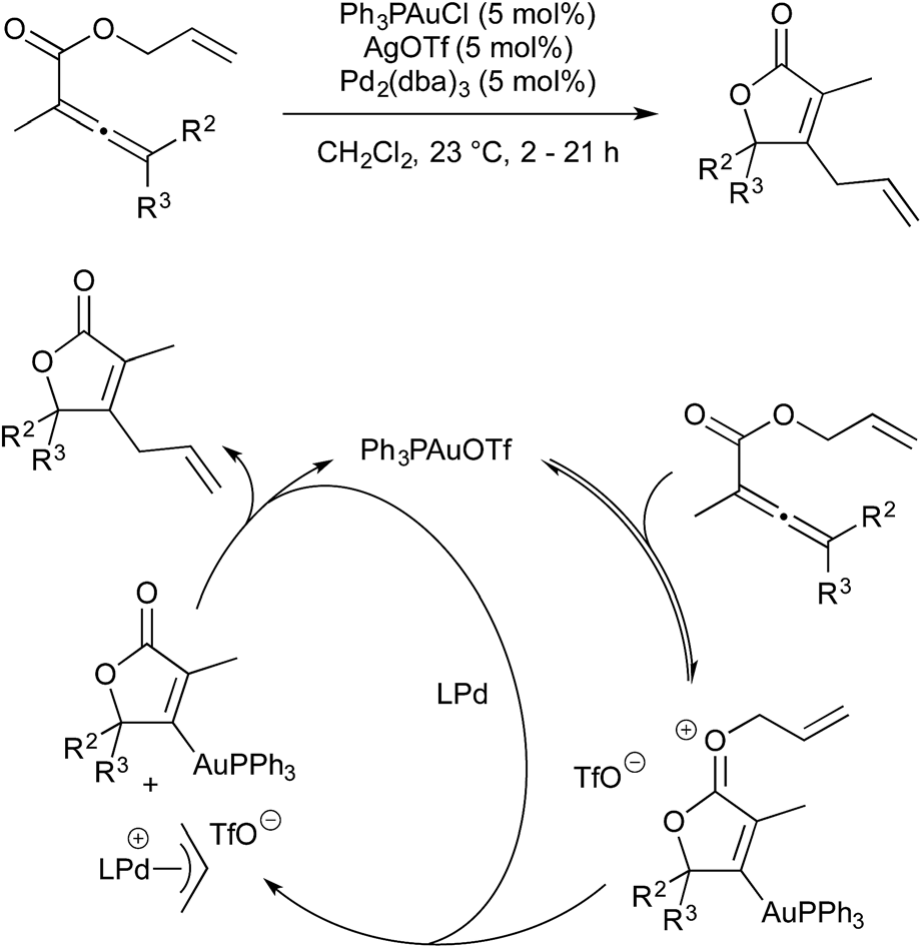
Au/Pd catalysis developed by Blum.

In 2009 Blum and co-workers published a gold/palladium-catalysed rearrangement of allyl allenoates to butenolides.^54^ The reaction proceeds smoothly at room temperature in the presence of an *in situ* generated gold(i) triflate complex and Pd_2_(dba)_3_ (5 mol% each) (Scheme 16). The initial step in the catalytic cycle is coordination of gold to the allene followed by cyclisation to give an activated gold–oxonium species. The allyl moiety is now activated towards oxidative addition by Pd(0) (oxidative addition does not occur in the absence of gold activation). Transmetallation from gold to palladium, followed by reductive elimination, give the butenolide product.

**Scheme 17:**
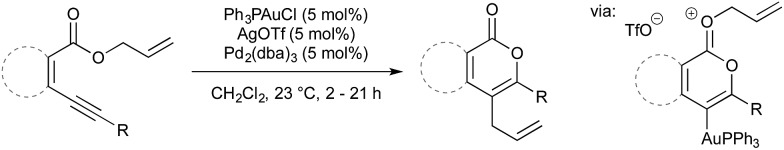
Au/Pd catalysis for lactone formation developed by Blum.

Included in the above publication was similar rearrangement of allyl esters onto alkynes to give lactones (Scheme 17). The scope of this rearrangement was expanded, and mechanistic details disclosed, in a 2014 report.^55^ It is worth noting that, in 2012, Hashmi showed that, when more activated esters are used as substrates (*i.e.* benzyl or cinnamyl in place of allyl) high yields of the products were obtained under monometallic gold-catalysed conditions.^56^

**Scheme 18:**
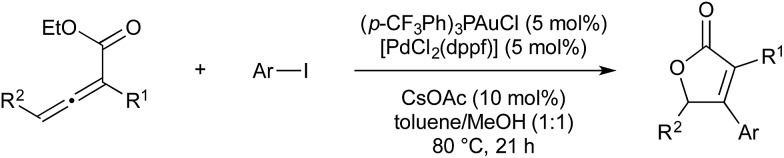
Au/Pd catalysis developed by Nevado.

In 2016 Nevado and co-workers published another rearrangement of allenoates; however in this report the gold-catalysed rearrangement is coupled with a palladium-catalysed aryl cross-coupling cycle (Scheme 18).^57^ Gold-catalysed carbocyclisation of the initial allenoate is followed by transmetallation to Pd(ii) aryl and reductive elimination.

**Scheme 19:**
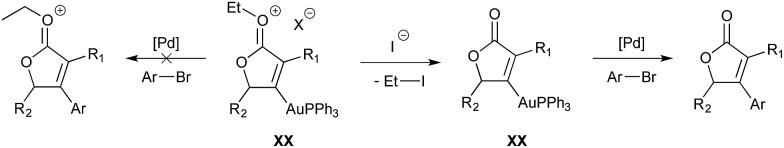
Au/Pd mechanistic results from Blum.

As part of the mechanistic investigations on allenoate rearrangements, Blum and co-workers noted that cationic oxonium species did not undergo palladium-catalysed cross-coupling, however the neutral analogue would.^54^ It is the judicious choice of aryl iodides as coupling partners that allow the Nevado reaction to proceed. The iodide formed on oxidative addition of Ar-I to Pd(0) acts as a dealkylating agent, giving iodoethane and a neutral butenolidyl gold species (Scheme 19). This reaction also sequesters iodide from the reaction medium, which is beneficial as Nevado reported that (*p*-F_3_CPh)_3_PAuI is inactive.

### 2.3 Others

**Scheme 20:**
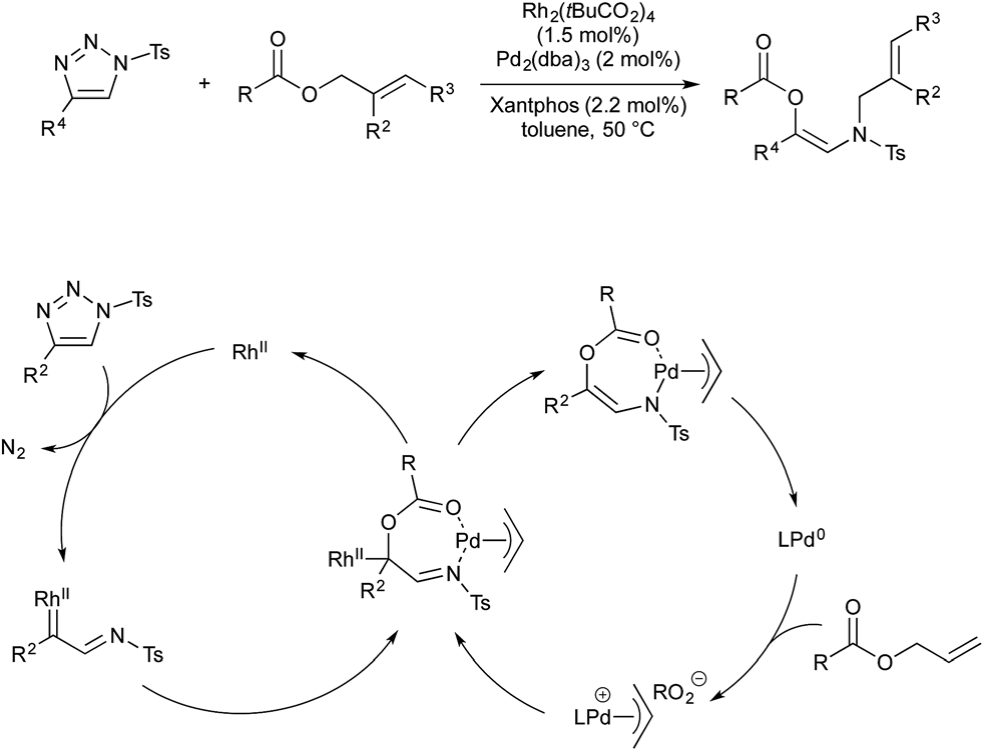
Rh/Pd catalysis developed by Lee.

Many other organometallic nucleophile cycles have been combined with Pd catalysis, following upon pioneering early examples.^58^,^59^ In 2016 Lee and co-workers demonstrated a rhodium–palladium-catalysed method that uses triazoles as precursors to rhodium-carbenes, which then couple with palladium-allyl complexes generated *in situ*.^60^ This procedure is unusual, in that the anion formed in oxidative generation of Pd-allyl is incorporated into the final product, increasing atom economy. Rhodium(ii) is known to activate triazoles to give α-iminocarbenoids. This underdoes nucleophilic attack by the carboxylate anion formed on oxidative addition of an allyl ester by Pd(0). The resultant imine-ester coordinates palladium in a seven-membered intermediate, from which rhodium(ii) is eliminated. Reductive elimination of the allyl fragment with *N*-tosyl furnishes the product and regenerates Pd(0) (Scheme 20).

**Scheme 21:**
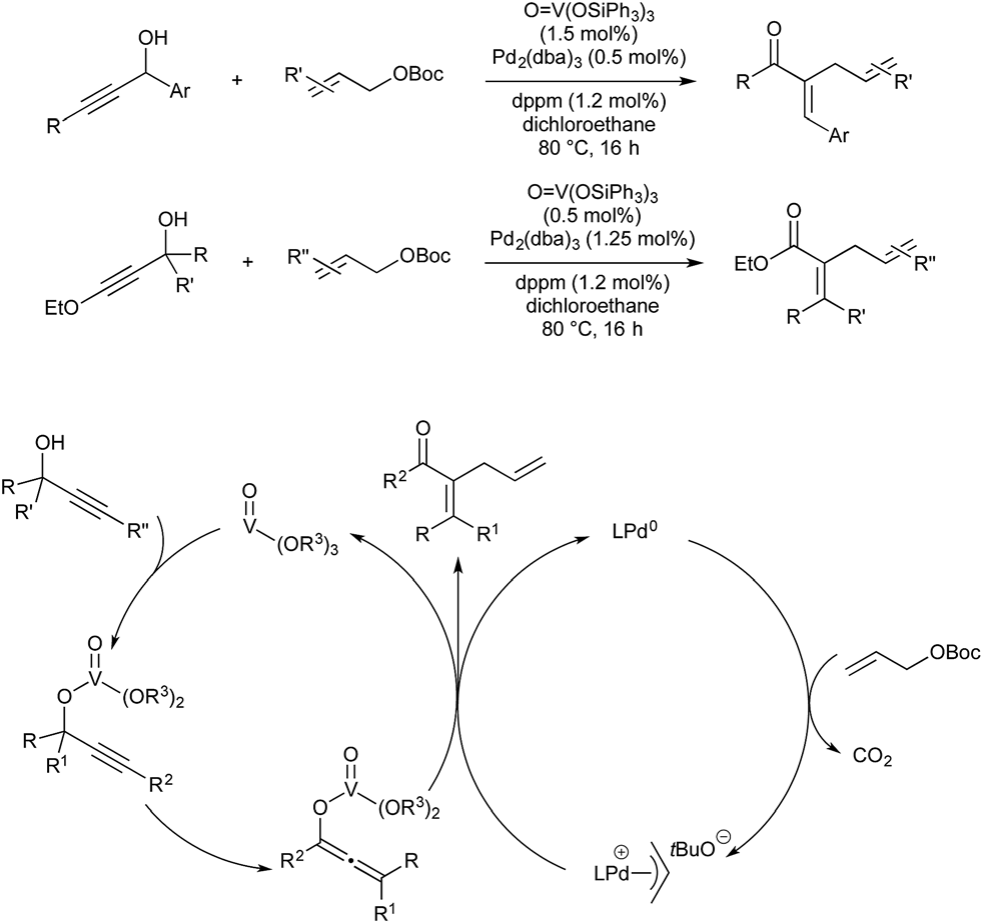
V/Pd catalysis developed by Trost.

Vanadium(v) esters are known to catalyse the rearrangement of propargylic alcohols *via* their corresponding vanadium allenolate. These can be trapped by various *N*-, *O*- or *C*-based nucleophiles. Trost and co-workers reported twice in 2011 on the interception of these intermediates with palladium-allyl complexes to give α-allyl-α,β-unsaturated ketones in a vanadium–palladium dual-catalysed process (Scheme 21). The first report details conditions for the use of aromatic propargylic alcohols.^61^ As they found that this aromatic activation was essential for the initial vanadium-catalysed rearrangement, later than year they expanded the scope to include aliphatic propargylic alcohols with an activating alkoxy group on the alkyne fragment.^62^

**Scheme 22:**
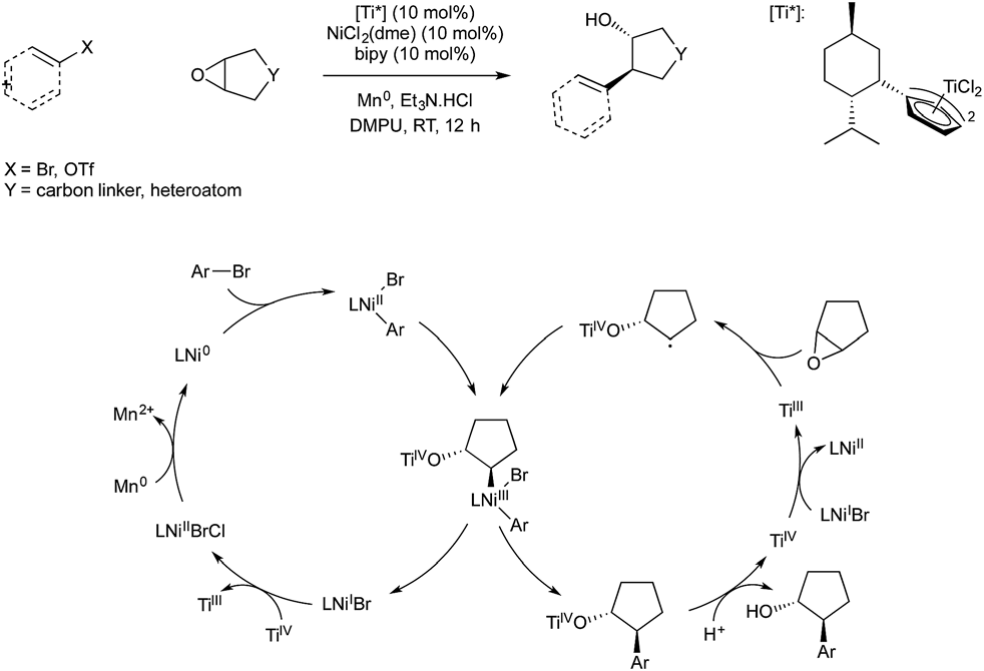
Ti/Ni catalysis developed by Weix.

Weix and co-workers demonstrated in 2014 that titanium(iii)-catalysed radical ring-opening of epoxides could be used in conjunction with reductive nickel catalysis to give an enantioselective coupling of epoxides and aryl bromides.^63^ The study utilised the fact that Ti(iii) is known to catalyse reductive ring opening of epoxides to give α-radical Ti(iv) alkoxides. Weix has shown in previous reductive cross-couplings that carbon centred radicals will react with arylnickel(ii) species to give Ni(iii) which rapidly undergoes reductive elimination. The resultant Ni(i) reduces Ti(iv) produced in epoxide ring opening to give Ni(ii) and regenerate Ti(iii), and Ni(ii) is in turn reduced to Ni(0) by manganese to complete both catalytic cycles (Scheme 22). The use of a chiral titanium precatalyst bearing Cp ligands derived from menthol allowed enantioselective ring opening of *meso*-epoxides, giving *trans* aryl-alcohols in high enantiopurity.

**Scheme 23:**
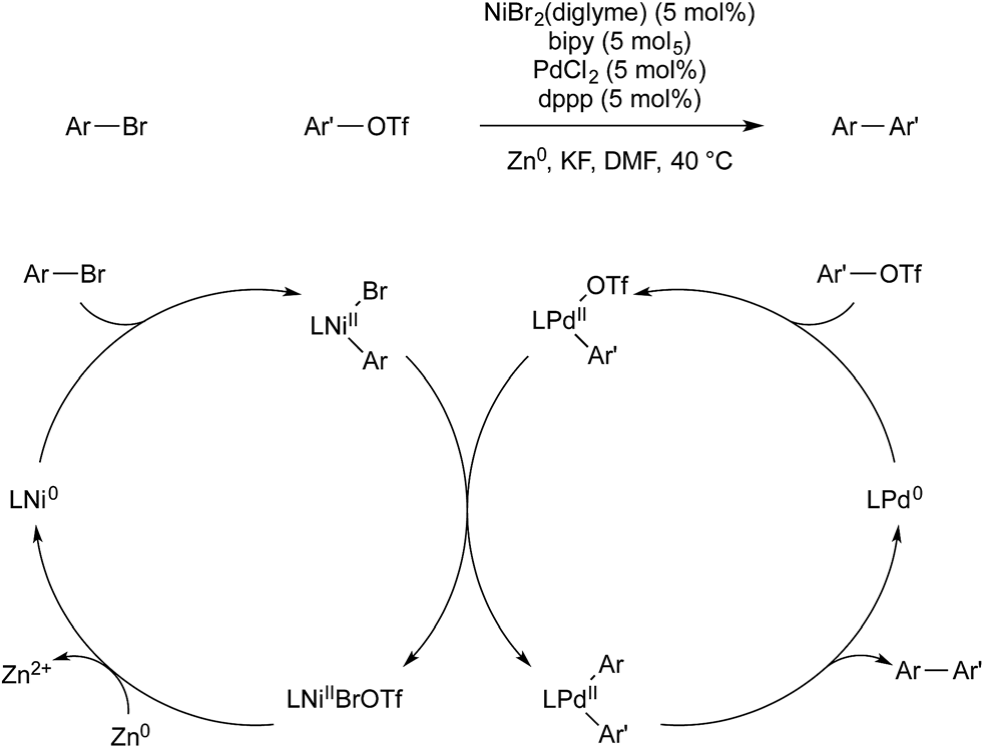
Ni/Pd catalysis developed by Weix.

Weix and co-workers have also used multimetallic catalysis for the reductive coupling of two aryl electrophiles.^64^ A 4,4′-bipyridine nickel precatalyst was employed for the activation of aryl bromides, and (dppp)PdCl_2_ for the activation of aryl triflates. Each monometallic catalyst reacts selectively with its intended aryl-electrophile, to give high selectivity for the cross-coupled product over the two possible homo-coupled side products. This selectivity also allows for the use of the electrophiles in a 1 : 1 ratio, whereas previous, monometallic, reductive cross-coupling of aryl halides had required excess of one reagent to obtain selectivity. After each metal undergoes oxidative addition of its respective electrophile, arylnickel(ii) transmetallates with arylpalladium(ii) to give bis(aryl)palladium(ii) intermediate, which reductively eliminates the desired product. Zinc metal is the terminal reductant, which regenerates nickel(0) and completes the catalytic cycle (Scheme 23).

**Scheme 24:**
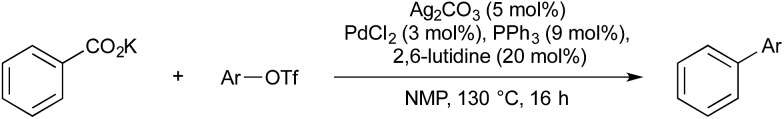
Ag/Pd catalysis developed by Goossen.

In 2010, Goossen and co-workers reported a silver–palladium-catalysed decarboxylative biaryl coupling (Scheme 24).^65^ Here, an organosilver intermediate is generated by silver-catalysed decarboxylation of a benzoic acid, and then employed as the nucleophilic partner for palladium-catalysed biaryl-coupling. This report is an extension of previous, analogous copper/palladium-catalysed reactions.^66^ Silver salts had previously been found to be more efficient in mediating the decarboxylation of benzoate salts, however they were found to be incompatible with cross-coupling conditions, as silver halide salts formed during the reaction would precipitate and prevent catalytic turnover. Goossen found that if aryl triflates were used as coupling partners, this salt precipitation was avoided and turnover achieved. Due to the greater efficiency of silver over copper to mediate decarboxylation, reaction temperatures could be lowered from 170 to 120 °C. Shen and coworkers have reported related Ag/Pd catalysis for difluoromethylation of aryl electrophiles.^67^

**Scheme 25:**
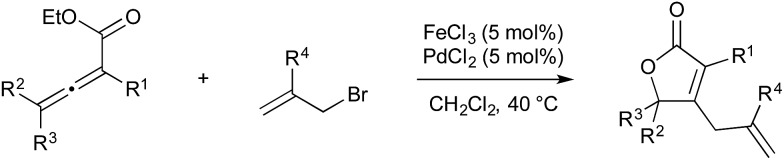
Fe/Pd catalysis developed by Chen and Ma.

In 2011 Chen and Ma published an iron/palladium-catalysed cyclisation/cross-coupling of allenoates with allyl electrophiles, which they claim is the first example of transmetallation of an organic fragment from iron to palladium (Scheme 25).^68^ The mechanism is similar to the gold/palladium-catalysed cyclisation/cross-coupling of allenoates published by Blum and Nevado; however in this instance iron(iii) chloride acts as the Lewis acidic metal, giving rise to formation of a metallo-lactone. Oxidative addition of allylic bromides to palladium(0) give electrophilic Pd(ii), which receives the lactone fragment from iron, and reductive elimination gives the allylic lactone as the product.

## 3. Bimetallic catalysis with binuclear bond breaking & forming mechanisms

### 3.1 Serendipitous bimetallic catalysis

While the dominant paradigm in homogeneous catalysis is to design reactions based on single-site mechanisms, in some cases a *post hoc* analysis of these reactions can reveal that two catalyst sites actually cooperate under the catalytic conditions even when bimetallic chemistry was unintended. Ideally, the elucidation of such serendipitous bimetallic reactions can be leveraged towards the rational design of bimetallic catalysts for useful transformations.

#### 3.1.1 Lewis acid catalysis

**Scheme 26:**
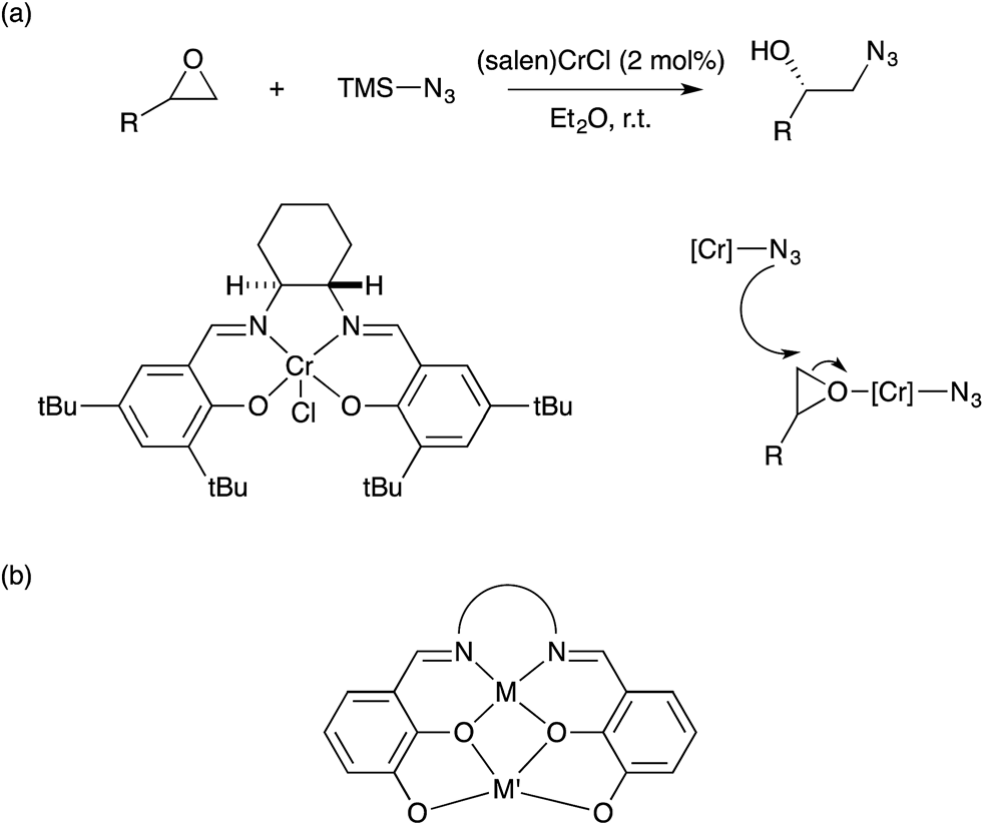
(a) Asymmetric epoxide ring-opening catalysis developed by Jacobsen; (b) heterobimetallic catalyst design of Shibasaki.

One prominent example of serendipitous bimetallic catalysis is the set of asymmetric epoxide ring-opening reactions advanced by Jacobsen.^69^ The initial design concept being pursued in these systems involved coordination of the epoxide substrate to a Lewis acidic Cr site buried within a chiral salen pocket, followed by stereospecific attack on the bound epoxide by an external nucleophile. However, the asymmetric ring-opening reaction involving azide as the nucleophile was found obey an experimentally-determined rate law with second-order dependence on the active catalyst, (salen)Cr(N_3_), thus implying the involvement of two catalyst molecules in the rate-determining step.^70^ The proposed model for this bimetallic step invokes intermolecular attack by a (salen)Cr(N_3_) nucleophile on an epoxide activated at a separate catalyst site (Scheme 26a). A similar model is operative for the unique regiodivergent ring-opening reactions of chiral aziridines developed by Parquette and RajanBabu using a related Y dimer where the two Lewis acidic catalyst sites are linked by bridging ligands.^71^ In addition to kinetics data, solid-state and solution-phase structural determination data are consistent with a rate-determining, intramolecular bimetallic step in this dimeric catalyst system.^72^ Shibasaki and others have produced many useful synthetic methods involving asymmetric reactions with related heterobimetallic Schiff base-ligated catalysts (Scheme 26b).^73^–^75^ These systems likely belong to the same mechanistic motif involving asymmetric bond formation induced by adjacent metal sites within a chiral pocket.

#### 3.1.2 Oxidative C–X coupling

**Scheme 27:**
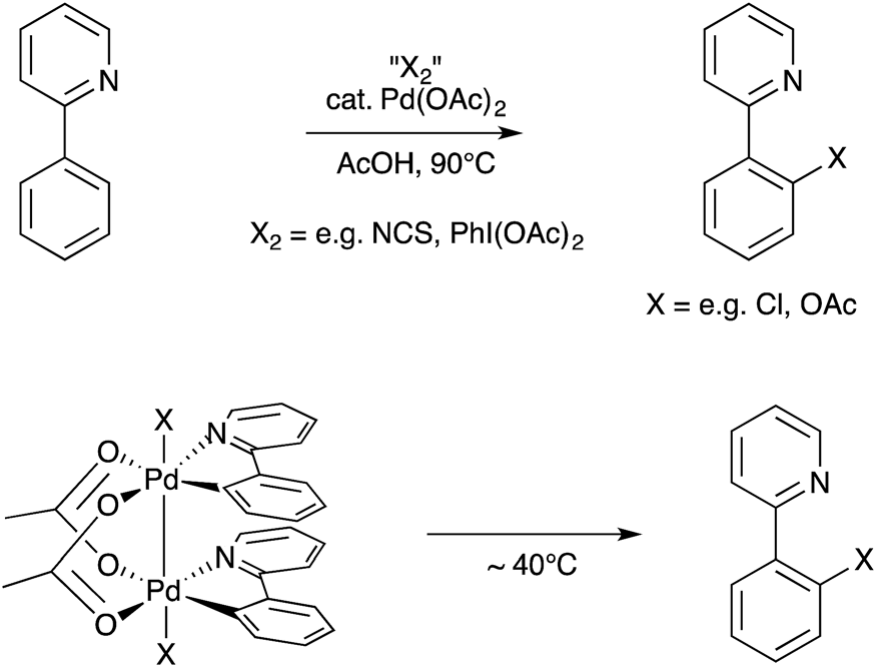
Oxidative catalysis involving dipalladium(iii) intermediates identified by Ritter.

Oxidative C–X bond formation catalyzed by Pd is often assumed to involve single-site Pd(ii)/Pd(iv) redox cycling.^76^ In 2009, Ritter and coworkers identified metal–metal bonded Pd(iii) intermediates that assembled under catalytic conditions and were competent at undergoing bimetallic reductive elimination of C–O and C–Cl bonds (Scheme 27).^77^ The nature of these bimetallic bond elimination reactions was subsequently analyzed in detail both experimentally and computationally.^78^ This discovery raised the possibility of bimetallic intermediates being involved in oxidative Pd catalysis and presented bimetallic Pd(ii)···Pd(ii)/Pd(iii)–Pd(iii) redox cycling (facilitated by bridging carboxylate ligands) as an alternative mechanistic manifold to the more traditional single-site Pd(ii)/Pd(iv) redox cycling model.^79^ Subsequent studies by Ritter, Sanford, Canty, Yates, and others have revealed that the bimetallic and single-site manifolds both are viable under typical reaction conditions,^80^ implying that partitioning between mononuclear and binuclear pathways needs to be evaluated on a case-by-case basis in such oxidative catalysis. Nonetheless, this new intellectual framework allowed for the rational development of a bimetallic Pd catalyst for C–H oxidation with O_2_ by Ritter.^81^

**Scheme 28:**
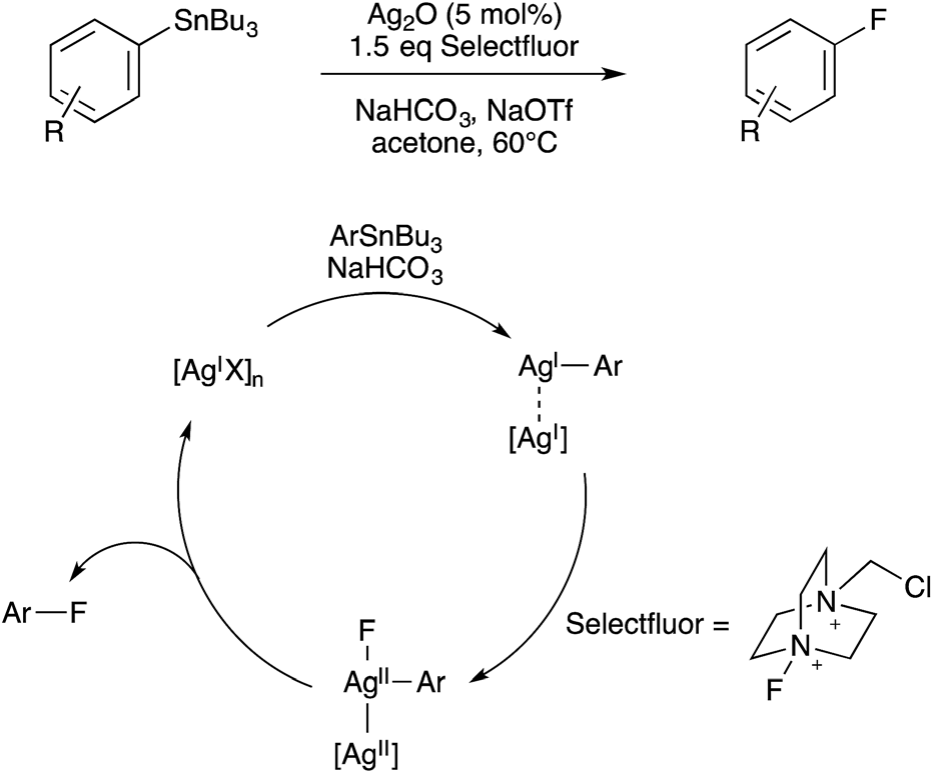
Bimetallic mechanism for Ag-catalyzed fluorination proposed by Ritter.

Furthermore, similar binuclear intermediates have come to be proposed in various coinage metal-catalyzed C–X bond forming oxidations, again raising the possibility of binuclear catalytic mechanisms being operative under certain conditions. First, in 2010, Ritter proposed that Ag(i)-catalyzed C–F coupling under oxidative conditions involves the intermediacy of bimetallic Ag(ii)–Ag(ii)–F species capable of binuclear C–F elimination,^82^ thus avoiding the invocation of high-valent Ag(iii)–F intermediates (Scheme 28). Indirect evidence for this bimetallic mechanism was obtained from the observed rate acceleration of C–F elimination from AgOTf additive during reaction of Selectfluor with isolated arylsilver(i) species.

**Scheme 29:**
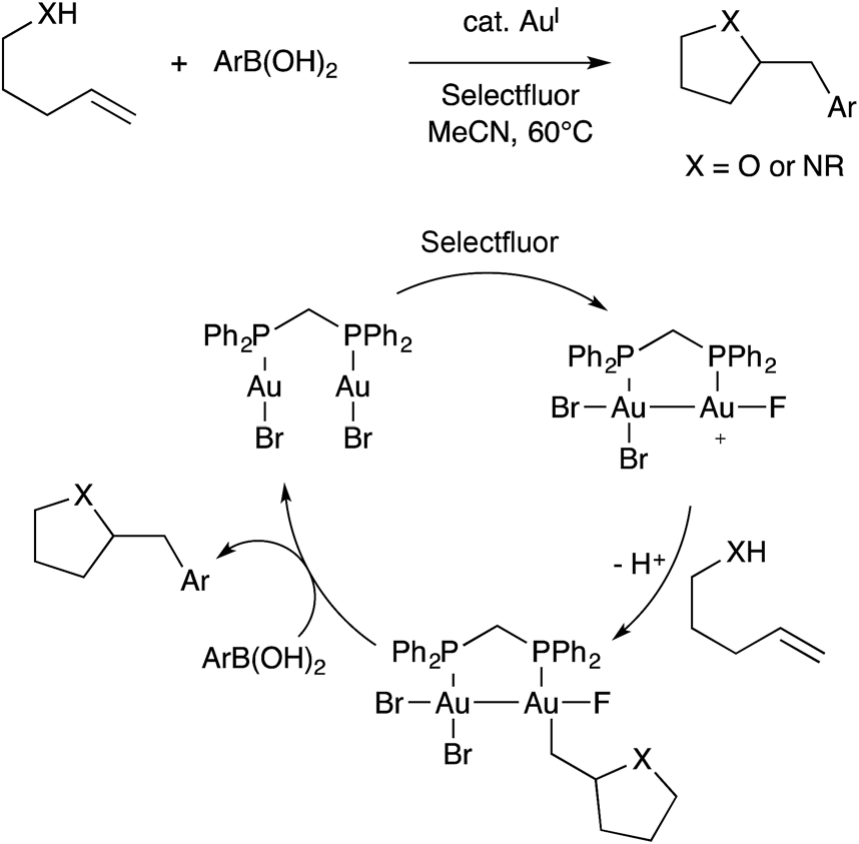
A representative example of oxidative Au catalysis, and the bimetallic mechanism proposed by Toste.

Contemporaneously, Zhang, Toste, Nevado, Gouverneur, Hashmi, Lloyd-Jones, and others developed oxidative alkene heteroarylation reactions using Au(i) catalysts combined with a stoichiometric oxidant, commonly Selectfluor. These reaction are typically proposed to involve single-site Au(i)/Au(iii) redox cycling, as was reviewed recently by Gouverneur.^83^ Among these various studies, both mononuclear and binuclear gold catalysts were employed. In one study, Toste found that the binuclear dppm(AuBr)_2_ catalyst exhibited superior performance when compared to mononuclear Ph_3_PAuBr.^84^ Furthermore, this effect disappeared when dppm was replaced with a longer and more flexible dppb tether. These observations led Toste and workers to propose a revised mechanism involving Au(ii)–Au(ii) intermediates capable of C–C coupling with arylboronic acid nucleophiles (Scheme 29).^85^ The bimetallic mechanism had support from computational studies. Additionally, cyclic voltammetry measurements were used to show that binuclear Au(i)···Au(i) aggregates featuring aurophilic interactions facilitated by bridging ligands are more readily oxidized than single-site Au(i) species lacking bridging ligands. Presumably the difference arises from stabilization of oxidized Au(ii)–Au(ii) species in comparison to high-valent Au(iii) species. Thus, under oxidative conditions, it is possible that bimetallic Au(ii)–Au(ii) intermediates assemble during catalysis even in the absence of bridging ligands.

In the Ag-catalyzed fluorination studied by Ritter and the Au-catalyzed heteroarylation studied by Toste, the binuclear coinage metal mechanisms serve to facilitate oxidative catalysis by providing a lower-barrier alternative to the high-potential M(i)/M(iii) redox couple. This concept is analogous to the binuclear Pd_2_ catalysis unveiled by Ritter. This intellectual framework led to the rational design of a binuclear Au(i) catalyst for C(sp^3^)–C(sp^2^) coupling with arylboronic acids developed by Toste.^86^

#### 3.1.3 Multicomponent coupling

**Scheme 30:**
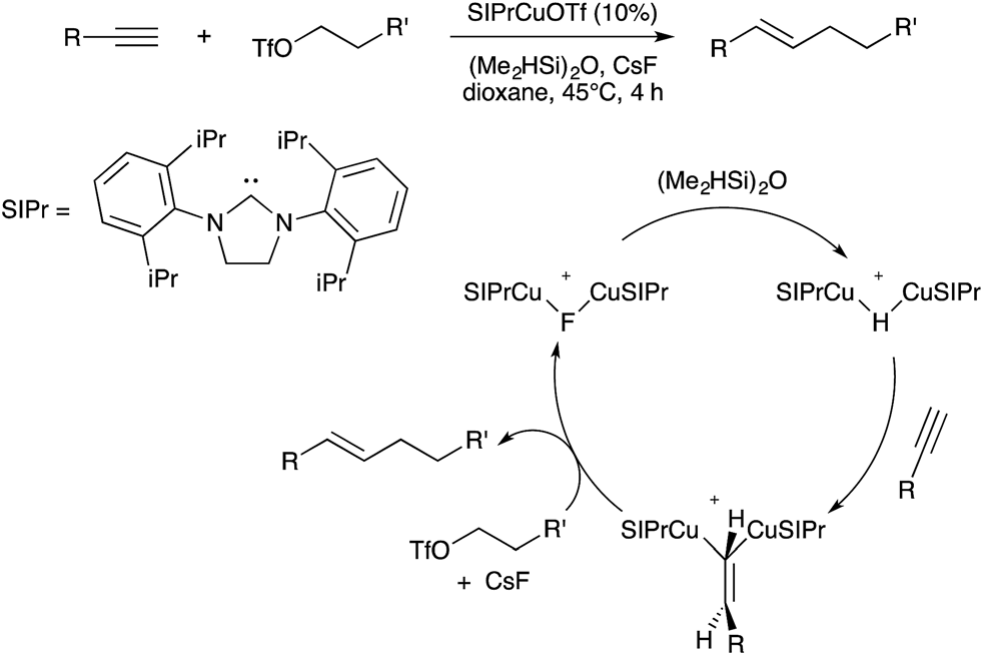
Hydroalkylation of alkynes developed by Lalic, and proposed binuclear catalytic mechanism.

In 2015, Lalic and coworkers disclosed a Cu-catalyzed hydroalkylation of terminal alkynes.^87^ The reaction conditions utilized a silane as the reductant, alkyl triflates as the electrophilic coupling partners, and fluoride as an additive to facilitate transmetallation. Although a single-site Cu mechanism was originally proposed, subsequent mechanistic studies led Lalic to revise the mechanism and propose binuclear intermediates throughout the catalytic cycle (Scheme 30).^88^ Stoichiometric reactivity studies indicated that the relevant mononuclear Cu intermediates would readily facilitate both reduction and fluorination of the alkyl triflate electrophile in competition with productive hydroalkylation, while binuclear Cu intermediates were shown to be less reactive towards these unproductive side processes. Because the catalytic conditions produced the hydroalkylation product in high yields without observation of unwanted alkyl reduction and fluorination products, it was proposed that the binuclear intermediates assemble as the active species during catalysis. Unlike the coinage metal chemistry described above, here binuclearity plays no role in facilitating redox cycling. Instead, the binuclear species here serve to attenuate the reactivity of hydride and fluoride intermediates during catalysis, thus imparting useful selectivity onto the multicomponent reaction.

### 3.2 Pre-assembled bimetallic catalysts

The examples in the previous section involve serendipitous bimetallic assembly under catalytic conditions. Beyond such examples, the rational design of bimetallic catalysis involving metal–metal bonds and/or bifunctional metal sites has long been a goal in the organometallic chemistry community. Historically, perhaps the most well-known set of bimetallic catalysts in organic synthesis are the dirhodium(ii) carboxylate paddlewheel complexes that catalyze various group transfer reactions that allow for C–C and C–N bond formation.^89^–^91^ In addition, the bimetallic species Co_2_(CO)_8_ is known to mediate C–C coupling reactions of alkynes such as the Pauson–Khand reaction, although stoichiometric Co is often required.^92^ Recent developments in these areas highlighted below indicate that pre-assembled bimetallic systems, often featuring metal–metal bonds with or without bridging ligands, are promising candidates for further catalyst development.

#### 3.2.1 Bifunctional catalysts

**Scheme 31:**
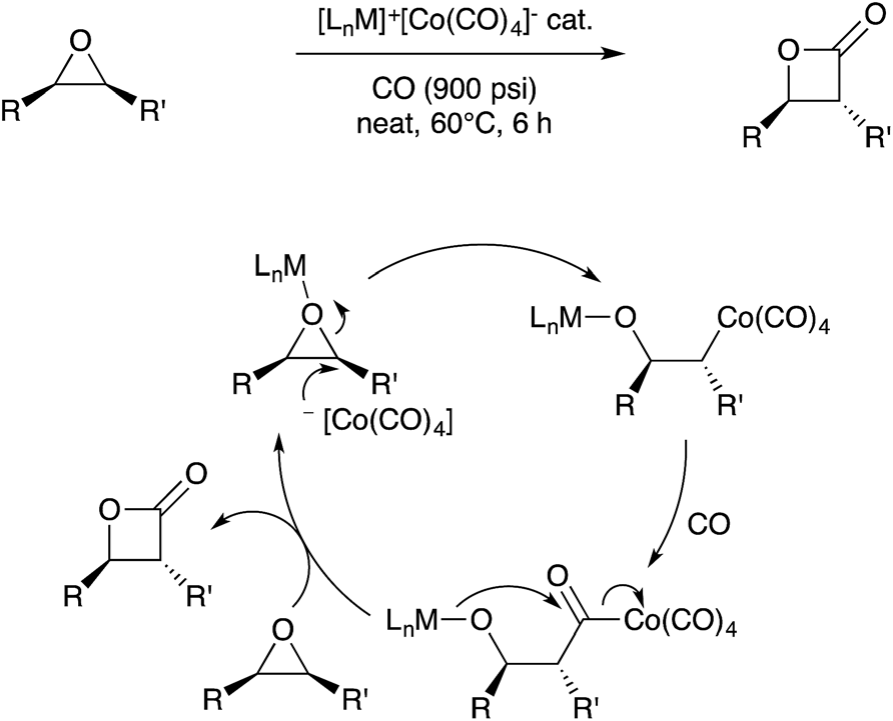
Bifunctional catalysis for epoxide carbonylation developed by Coates.

In analogy to the emergence of frustrated Lewis pairs in metal-free catalysis,^93^ a powerful concept in transition metal catalysis is to harness the bifunctional reactivity of Lewis acid/base bimetallic pairs.^94^ A particularly illustrative example of this approach was reported by Coates and coworkers in 2005.^95^ A bimetallic catalyst consisting of [(OEP)Cr(THF)_2_]^+^ and [Co(CO)_4_]^–^ as a separated ion pair was used to catalyse the stereospecific carbonylation of epoxides to β-lactones (OEP = octaethylporphyrinate). In related studies the cationic Cr fragment has been replaced with a variety of metal and non-metal motifs such as [(salen)Al]^+^ cations, even allowing for enantioselective variants to emerge.^96^,^97^ For all cases, a bimetallic mechanism is proposed (Scheme 31) wherein both metal sites cooperate to ring-open the epoxide substrate. Upon carbonylation of the resultant alkylcobalt intermediate, the bifunctional nature of the system allows for product release by ring-closing lactonization.

**Scheme 32:**
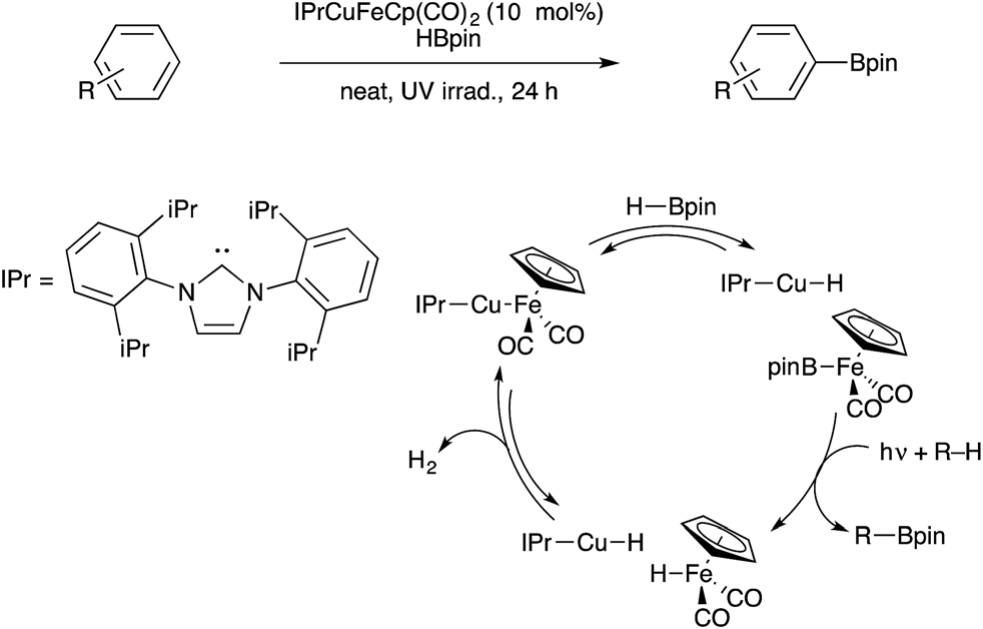
Bifunctional catalysis for C–H borylation developed by Mankad.

Beginning in 2013, our group began studying bifunctional catalysts closely related to these heterobimetallic ion pairs. A series of (NHC)Cu–[M_CO_] catalysts, including derivatives with [M_CO_] = Co(CO)_4_, were characterized (NHC = N-heterocyclic carbene).^98^ Although these complexes do feature Cu–M bonds in the solid state, theoretical analysis indicates that these copper–metal bonds are highly polarized donor/acceptor-type dative interactions. Furthermore, crossover experiments are consistent with equilibrium concentrations of [(NHC)Cu]^+^[M_CO_]^–^ pairs forming in solution.^99^ One derivative, a (NHC)Cu–FeCp(CO)_2_ species, was shown to catalyse dehydrogenative borylation of unactivated arenes upon photochemical activation (Scheme 32).^100^ Based on stoichiometric reactivity studies and computational analysis,^99^ it was proposed that the bifunctional Cu/Fe catalyst reacts with the boron source to generate CpFe(CO)_2_(Bpin) as the active borylating species (HBpin = pinacolborane). An analogous boryliron complex had previously been shown by Hartwig to mediate stoichiometric C–H borylation.^101^ The bifunctional Cu/Fe pair is thought to be crucial for cleaving the B–H bond of HBpin in order to generate the active borylating intermediate. Upon C–H borylation, the Cu/Fe catalyst is regenerated through a binuclear H_2_ elimination that proceeds through a highly polar transition state.^102^ The bifunctional nature of this series of catalysts is actively being pursued for a variety of other catalytic applications.^21^,^30^

#### 3.2.2 Persistent binuclear catalysts

**Scheme 33:**
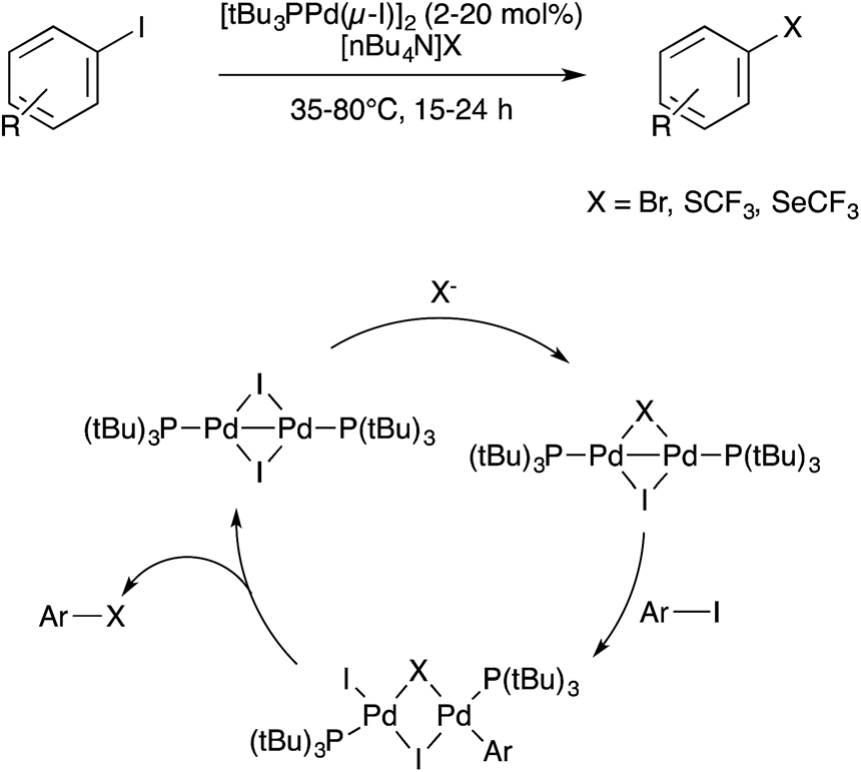
Pd(i)-catalyzed C–X coupling developed by Schoenebeck.

Pd catalysis is dominated by single-site oxidative addition/reductive elimination mechanisms involving Pd(0)/Pd(ii) redox cycling. Under oxidative conditions, Ritter has shown that bimetallic intermediates assemble under certain reaction conditions, as discussed above. However, very little bimetallic Pd catalysis has been developed through rational design for C–C or C–X coupling purposes. Schoenebeck and coworkers have recently reported a series of studies indicating that robust metal–metal bonded Pd(i)–Pd(i) dimers are promising candidates that have reactivity complementary to standard Pd(0) catalysts. In their initial foray in 2013, Schoenebeck and coworkers definitively showed that the dinuclear Pd(i) catalyst [*t*Bu_3_Pd(μ-I)]_2_, and not any mononuclear Pd(0) species that might form from reduction or disproportionation, is the active catalyst for an unusual I-for-Br aryl halide exchange reaction.^103^ Subsequently, Pd(i)-catalysed C–S and C–Se coupling reactions were reported using the same approach.^104^,^105^ All three of these transformations are proposed to proceed by analogous bimetallic mechanisms that involve Pd(i)–Pd(i)/Pd(ii)···Pd(ii) redox cycling (Scheme 33). In addition to the complementary reactivity exhibited by these Pd(i) dimers, advantages of this approach include operational simplicity and facile recyclability due to the air- and moisture-stability of the Pd(i) catalysts.

**Scheme 34:**
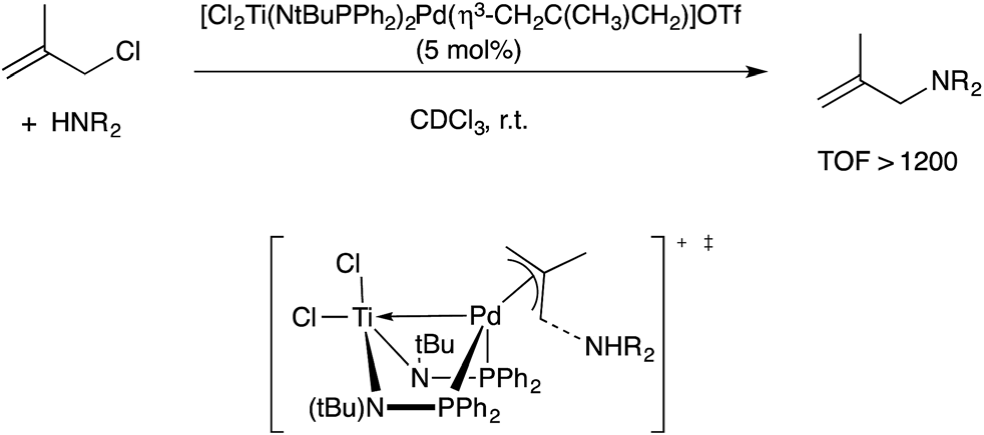
Allylic amination-catalysed by a Pd–Ti heterobimetallic complex, and the proposed rate-determining C–N coupling transition state proposed by Michaelis and Ess.

In another Pd-catalyzed C–X coupling process, Nagashima reported that the heterobimetallic cation [Cl_2_Ti(NtBuPPh_2_)_2_Pd(η^3^-CH_2_C(CH_3_)CH_2_)]^+^ was capable of extremely efficient allylic amination catalysis.^106^ Michaelis, Ess, and coworkers conducted a combined experimental/computational mechanistic study on the empirically observed rate acceleration compared to monometallic Pd-allyl cation catalysts.^107^ A model for rate-determining C–N bond formation was proposed, with the dative Pd → Ti interaction stabilizing Pd(0) character in the transition state and therefore lowering the reductive elimination barrier (Scheme 34). When compared to appropriate monometallic counterparts, rate enhancements of up to 10^5^ were documented in some cases. A Co/Zr-catalyzed Kumada coupling reaction studied by Thomas and coworkers is likely another example of this mechanistic motif.^108^

**Scheme 35:**
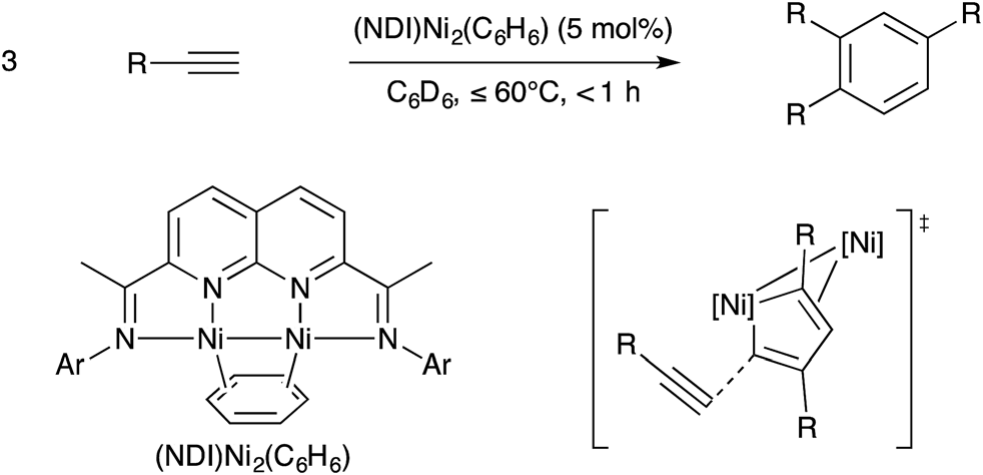
Dinickel-catalysed alkyne cyclotrimerisation developed by Uyeda, and the proposed bimetallic transition state that steers regioselectivity.

In part due to the known reactivity of the metal–metal singled bonded Co_2_(CO)_8_ towards alkynes and in part due to the potential of using metal–metal multiple bonds to engage unsaturated substrates in pericyclic reactions, bimetallic catalysts have long been examined for catalytic alkyne cyclotrimerisation reactions. This topic was reviewed thoroughly by Mashima recently.^109^ Among the most exciting advances is a bimetallic Ni catalyst supported by a binucleating naphthyridine-diimine (NDI) ligand that Uyeda and coworkers reported in 2015 is effective at selective and efficient alkyne cyclotrimerisation.^110^ The metal–metal bonded binuclear catalyst was highly active for coupling terminal alkynes to provide 1,2,4-substituted arene products selectively in preference to 1,3,5-substituted regioisomers or cyclotetramers. On the other hand, related mononuclear Ni catalysts supported by bipyridine, diimine, or pyridylimine ligands were significantly less active and gave complex product mixtures. Based on stoichiometric model reactions, a mechanism was proposed by which alkyne dimerization occurs to yield a nickelacyclopentadiene intermediate, which subsequently couples with a third alkyne equivalent to release arene. The terminal arene-releasing reaction was proposed to be the key selectivity-determining step. Crystallographic and theoretical evidence based on model intermediates was consistent with the cyclic fragment located at the reactive Ni site engaging in a secondary π-interaction with the spectator Ni site. This secondary π-interaction was proposed to stabilise one C=C bond and thereby direct the third alkyne substrate towards the uncoordinated C=C bond (Scheme 35). Approach of the least hindered carbon centre to this reactive C=C moiety thus provides the observed 1,2,4-substituted regioisomer.

## 4. Conclusions

In conclusion, bimetallic catalysis allows for novel modes of C–C and C–X bond formation to occur. Rate enhancements, selectivity control, and/or non-precious metal chemistry have all been enabled by bimetallic strategies, as summarized in this perspective. A large portion of the bimetallic catalysis literature as applied to C–C and C–X bond formation involves interfacing classical Pd catalysis with catalytically generated organometallic nucleophiles. Furthermore, at this time the preponderance of such examples involve organocopper nucleophiles and therefore descend from the seminal Sonogashira coupling reaction. Catalytic transformations that employ binuclear bond activation/formation steps, particularly by rational design, are comparatively underdeveloped and ripe for exploration.
